# Obesity drives the link between liver fat and depression: Cross‐sectional and prospective investigations

**DOI:** 10.1111/dom.16562

**Published:** 2025-06-24

**Authors:** Qi Feng, Chioma N. Izzi‐Engbeaya, Pinelopi Manousou, Mark Woodward

**Affiliations:** ^1^ The George Institute for Global Health (UK), School of Public Health, Faculty of Medicine Imperial College London London UK; ^2^ Section of Investigative Medicine and Endocrinology, Department of Metabolism, Digestion and Reproduction, Faculty of Medicine Imperial College London UK; ^3^ Department of Endocrinology St Mary's Hospital, Imperial College Healthcare NHS Trust London UK; ^4^ Division of Digestive Diseases, Department of Metabolism, Digestion and Reproduction, Faculty of Medicine Imperial College London London UK; ^5^ Department of Hepatology St Mary's Hospital, Imperial College Healthcare NHS Trust London UK; ^6^ The George Institute for Global Health (Australia) University of South New Wales Sydney Australia

**Keywords:** depression, liver fat, obesity, steatotic liver disease

## Abstract

**Aims:**

Inconsistent associations have been reported between steatotic liver disease (SLD) and depression. We aimed to investigate the cross‐sectional and prospective associations between MRI‐derived liver fat, SLD and depression.

**Materials and Methods:**

We used UK Biobank data. Liver fat was measured with liver MRI proton density fat fraction. SLD was defined as liver fat ≥5%. Depression was identified through self‐reported diagnosis and hospital records. We examined cross‐sectional associations using logistic regression and prospective associations using Cox proportional hazards models, adjusting for potential confounders, including socioeconomic status, lifestyle factors and body mass index (BMI).

**Results:**

Participants numbering 36 587 were included (age 64.5 years, 51.4% females, 28.2% SLD), and of these, 2849 participants had prevalent depression, higher in individuals with SLD (9.1%) than in those without (7.3%). SLD was associated with 40% higher odds of depression (OR: 1.40 (1.29, 1.52)) after adjusting for age, sex and socioeconomic status, but this was attenuated (OR: 1.12 (1.01, 1.23)) after adjusting for BMI. In the prospective analysis (*n* = 33 762), 414 people received a new diagnosis of depression over a median follow‐up of 4.5 years. SLD was initially associated with a 27% higher depression risk (HR: 1.27 [1.03, 1.56]), but this was no longer significant after BMI adjustment (HR: 0.93 (0.73, 1.18)). Liver fat (per 5% increase) was not associated with depression risk (HR: 1.02 (0.92, 1.12)). No sex differences were identified.

**Conclusions:**

The association between liver fat and depression is likely due to reverse causation and the confounding effect of BMI.

## 1 INTRODUCTION

Steatotic liver disease (SLD) is the most prevalent chronic liver disease, affecting nearly 33% of adults worldwide.^1^ SLD is characterized by excessive liver fat (>5%) and is strongly linked to cardiometabolic risk factors (CMRF).^2^, ^3^ SLD encompasses three subtypes, depending on the alcohol consumption level and presence of CMRFs: metabolic dysfunction associated steatotic liver disease (MASLD), metabolic dysfunction and alcohol‐related steatotic liver disease (MetALD) and alcohol‐related liver disease (ALD).^2^, ^4^ SLD is linked to elevated inflammatory markers, such as C‐reactive protein, triglyceride‐based indices and systemic immune‐inflammation index.^5^, ^6^, ^7^ SLD‐associated CMRFs, such as obesity, diabetes and broad metabolic syndrome, are also linked with increased chronic inflammation.^8^, ^9^, ^10^, ^11^ SLD may progress to cirrhosis, liver decompensation and hepatocellular carcinoma.^12^ In addition to hepatic complications, SLD has been associated with various extrahepatic diseases, such as extrahepatic cancers, cardiovascular disease and chronic kidney disease.^13^, ^14^, ^15^

Recent studies have also highlighted a high prevalence of depression among individuals with SLD. A systematic review of 31 studies involving 2 million adults with MASLD^16^ reported a prevalence of 26.3% for depression. Chronic inflammation is also considered associated with depression.^17^ However, findings on the association between SLD and depression remain conflicting. Early systematic reviews suggested a positive association between MASLD and depression, but all of the included studies were cross‐sectional,^18^, ^19^ prone to residual confounding and reverse causation. A recent prospective cohort study in Sweden similarly found a positive association between MASLD and depression and anxiety.^20^ By contrast, a study in Italy^21^ reported that the association between MASLD and depression was attenuated to null after adjusting for socioeconomic status and body mass index (BMI), aligning with Mendelian randomization evidence suggesting a lack of causal association.^22^ However, this Italian study had notable limitations, including its cross‐sectional design, small sample size (*n* = 286) and reliance on a single self‐reported depression questionnaire, rather than more accurate clinical diagnosis.

Despite these studies, the relationship between liver fat quantification and depression has not been investigated. Examining liver fat as a continuous measure could provide valuable insights into understanding the relationship between steatosis and depression. Therefore, this study aimed to assess the association between liver fat, SLD, SLD subtypes and depression. We examined both cross‐sectional associations with prevalence of depression, and prospective associations with incidence of depression.

## 2 MATERIALS AND METHODS

### 2.1 Participants

We used data from the UK Biobank, a prospective cohort of 0.5 million individuals aged 40–70 years, recruited between 2006 and 2010. Baseline data were collected on socioeconomic status, medical history, lifestyle factors and anthropometric data. Biological samples including blood, urine and saliva were also collected. Between 2010 and 2013, about 20 000 participants underwent a repeat assessment. Since 2014, UK Biobank has invited the participants to attend imaging visits, where magnetic resonance imaging (MRI) scans of the brain, heart and abdominal organs (including the liver, kidney and pancreas) were conducted. Up to February 2025, liver MRI data are available for ~40 000 participants. We excluded people who withdrew their consent, and those with missing data required to define SLD and subtypes.

### 2.2 Exposure assessment

Liver MRI images were acquired using a Siemens MAGNETOM Aera1.5 T scanner (Syngo MR D13) and the LiverMultiScan protocol from Perspectum Ltd. (UK), as part of the UK Biobank abdominal imaging protocol.^23^, ^24^ Liver fat percentage was estimated using proton density fat fraction (PDFF).

SLD was defined as PDFF ≥ 5.0%. Subtypes of SLD were classified into MASLD, MetALD, ALD and others, based on the definitions proposed by Rinella et al.,^2^ incorporating average daily alcohol intake and the presence of CMRFs. CMRFs, including obesity, diabetes, hypertension, high triglycerides and low high‐density lipoprotein (HDL) cholesterol, were also identified based on the definition proposed by Rinella et al.,^2^ using self‐reported diagnosis, hospital records, blood pressure measurement, anthropometric measurement (BMI and waist circumference (WC)), use of antihypertensive drugs, antidiabetic drugs and dyslipidaemia drugs, and blood biochemistry assessments (glycated haemoglobin (HbA1c), triglycerides (TG) and HDL). MASLD, MetALD and ALD were defined by presence of liver steatosis and CMRFs, and average daily alcohol intake of <20/30 (female/male), 20/30–50/60, ≥50/60 g/day, respectively. For biochemistry assessments, we used all available records from baseline and repeat assessments, because biochemistry data on the date of the imaging visit assessment are not available. Other data sources were based on imaging visit assessments for the most up‐to‐date information.

### 2.3 Outcome assessment

The outcomes were prevalent and incident depression diagnoses. Participants were followed up via linkage to the national death registry and hospital records. Depression was identified using self‐reported depression diagnoses at the imaging visit and hospital medical records, using International Classification of Diseases version 10 (ICD10) codes F32‐F33.

### 2.4 Covariates

Ethnicity was classified into White and others. The Townsend Deprivation Index is a postcode‐derived measure used to designate socioeconomic status. Highest educational attainment was categorized as below secondary, lower secondary, upper secondary, vocational training and higher education. Lifestyle factors considered were self‐reported smoking status (current, previous and never smoker), daily alcohol consumption (g/day), and physical activity level. Physical activity level was measured with the International Physical Activity Questionnaire and categorized into low, moderate and high levels. Systolic and diastolic blood pressure were measured twice within a few minutes apart and the averages of the two readings were used in analyses. Blood biochemistry markers were measured at a central laboratory.^25^ Anthropometric measures included BMI, body fat percentage (BF%) and central obesity measures, including WC, waist‐to‐hip ratio (WHR), waist‐to‐height ratio (WHtR). BF% was measured with bioimpedance analyser. FIB4 score was a non‐invasive assessment for fibrosis status,^26^ calculated at baseline assessment. For all the categorical covariates, answers of ‘unknown’, ‘do not know‘, ‘prefer not to say’ were combined into one ‘unknown’ category.

### 2.5 Statistical analysis

For descriptive statistics, we used mean with standard deviation for normally distributed continuous variables, median with interquartile interval (IQI) for other continuous variables, and frequency with proportion for categorical variables. We assessed the correlation between PDFF and BMI using Spearman correlation coefficients, with *p* value of 0.05 for statistical significance level.

For cross‐sectional analysis, we used logistic regression to examine the associations between liver fat (continuous, per 5% increase), SLD and SLD subtypes with prevalent depression. We fitted a basic model adjusted for age, sex, ethnicity, education, Townsend Deprivation Index (in fifths) and further adjusted for smoking, physical activity, alcohol intake, FIB4 score and BMI progressively in various models. We also conducted sex‐stratified analyses to assess potential sex differences.

For prospective analysis, we excluded individuals with prevalent depression at baseline. The outcome was incident depression, censored at date of diagnosis, death or the last day of follow‐up (30 October 2022). We fitted Cox proportional hazard models to examine the association between liver fat, SLD and SLD subtypes with incidence of depression, with the same covariate adjustments as in the cross‐sectional analyses. The proportional hazard assumption was checked with scaled Schonfeld residuals, and no evidence for violation was observed.

Odds ratio (OR) and hazard ratio (HR) with their 95% confidence intervals (CIs) were used as the effect measures for cross‐sectional and prospective associations, respectively.

To assess for a non‐linear association between liver fat (continuous) and risk of depression, we also modelled liver fat using restricted cubic spline function with four knots. Furthermore, we assessed the association between liver fat severity (<5%, 5%–10%, and ≥10%) and depression. We conducted sex‐specific analyses and tested for sex interactions. We also performed other subgroup analyses, stratified by age group, Townsend Deprivation Index (fifths), education, smoking status, physical activity level and SLD status and subtypes, and tested for subgroup difference. For sensitivity analysis, we (1) excluded the first two years of follow‐up, to correct for reverse causation, (2) used alternative obesity measures to BMI, including WC, WHR, WHtR and BF%.

All analyses were performed in R (version 4.4.1).

## 3 RESULTS

**FIGURE 1:**
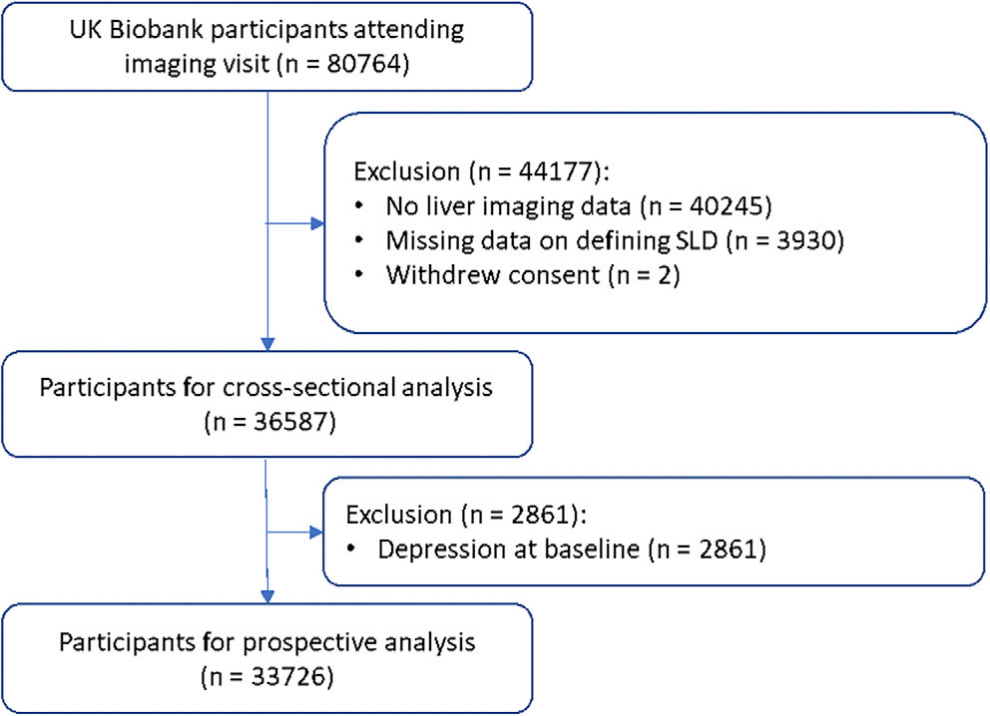
Flowchart of participant selection.

Among 40 519 participants who underwent liver imaging, 36 587 (age 64.5 years, females 51.4%) participants were included in the analysis (Figure 1). Of these, 10 304 (28.2%) had SLD, comprising 7791 (21.3%) with MASLD, 1838 (5.0%) with MetALD and 501 (1.4%) with ALD.

Compared with participants without SLD, participants with SLD were more likely to be males, from more deprived socioeconomic backgrounds, less well educated, ever smokers, physically inactive, and have higher alcohol intake, BMI and blood pressure. They were also more likely to have CMRFs (Table 1). The Spearman correlation between liver fat and BMI was 0.60 (*p* < 0.05). The median liver fat for people with MASLD, MetALD and ALD was 8.6 (IQI 6.3, 13.2), 8.6 (6.4, 13.2) and 9.7 (6.8, 16.1)%, respectively. More details on the characteristics of SLD subtypes are presented in supplementary Table S1.

**TABLE 1:** Baseline characteristics of participants with and without steatotic liver disease.

	Non SLD	SLD	Overall
	*n* = 26 283	*n* = 10 304	*n* = 36 587
Sex, female	14 766 (56.2%)	4028 (39.1%)	18 794 (51.4%)
Age, years	64.5 (7.7)	64.7 (7.4)	64.5 (7.7)
Townsend deprivation index			
1st fifth (least deprived)	5351 (20.4%)	1964 (19.1%)	7315 (20.0%)
5th fifth (most deprived)	5082 (19.3%)	2203 (21.4%)	7285 (20.0%)
Education, higher education	13 450 (51.2%)	4358 (42.3%)	17 808 (48.7%)
Ethnicity, White	25 466 (96.9%)	9969 (96.7%)	35 435 (96.9%)
Smoking, never	16 901 (64.3%)	5985 (58.1%)	22 886 (62.6%)
Alcohol intake, g/day	13.1 (13.9)	17.7 (20.2)	14.4 (16.1)
Physical activity, high	11 193 (42.6%)	3379 (32.8%)	14 572 (39.8%)
Liver fat, %	2.5 (2.0, 3.3)	8.6 (6.3, 13.3)	3.1 (2.2, 5.5)
Body mass index, kg/m^2^	25.2 (3.6)	29.6 (4.4)	26.5 (4.3)
Waist circumference, cm	84.6 (11.0)	97.6 (11.3)	88.3 (12.6)
Waist‐to‐hip ratio, %	85.5 (8.3)	92.8 (7.8)	87.5 (8.8)
Waist‐to‐height ratio, %	50.1 (6.0)	57.3 (6.4)	52.2 (6.9)
Body fat, %	29.8 (8.0)	33.6 (7.8)	30.9 (8.1)
Systolic blood pressure, mmHg	136.7 (18.5)	144.1 (17.5)	138.7 (18.6)
Diastolic blood pressure, mmHg	77.4 (9.9)	82.0 (9.7)	78.7 (10.0)
Hypertension	20 113 (76.5%)	9431 (91.5%)	29 544 (80.7%)
Obesity	14 409 (54.8%)	9396 (91.2%)	23 805 (65.1%)
Diabetes	2789 (10.6%)	2288 (22.2%)	5077 (13.9%)
High triglycerides	8390 (31.9%)	5991 (58.1%)	14 381 (39.3%)
Low HDL‐cholesterol	7643 (29.1%)	4118 (40.0%)	11 761 (32.1%)
SLD subtypes			
MASLD	NA	7791 (75.6%)	7791 (21.3%)
MetALD	NA	1838 (17.8%)	1838 (5.0%)
ALD	NA	501 (4.9%)	501 (1.4%)
Others	NA	174 (1.7%)	174 (0.5%)

*Note*: Mean with standard deviation for normally distributed continuous variable, median with interquartile interval for other continuous variables, and frequency with proportion for categorical variables.

Abbreviations: ALD, alcohol‐related liver disease; HDL, high‐density lipoprotein; MASLD, metabolic dysfunction‐associated steatotic liver disease; MetALD, metabolic dysfunction and alcohol‐related liver disease; NA, not applicable; SLD, steatotic liver disease.

### 3.1 Cross‐sectional associations

The results of cross‐sectional analyses are shown in the upper panel of Table 2. At baseline, 2849 participants had depression, with a prevalence of 7.3% (1923/26 283) in those without SLD and 9.1% (938/10 304) in those with SLD. In the basic model, adjusting for age, sex and socioeconomic status, SLD was associated with a 40% higher prevalence of depression (odds ratio [OR] [95% confidence interval]: 1.40 [1.29, 1.52]). Additionally, adjusting for smoking, physical activity and alcohol intake slightly attenuated the association (1.34 [1.23, 1.46]). However, further adjustment for BMI substantially reduced the association (1.12 [1.01, 1.23]). Among SLD subtypes, MASLD (1.12 [1.01, 1.25]) showed a similar pattern to overall SLD, while MetALD (1.04 [0.85, 1.27]) and ALD (1.09 [0.73, 1.62]) showed almost null associations, probably due to small sample size in these subtypes.

**TABLE 2:** Cross‐sectional and prospective associations between liver fat, SLD, SLD subtypes and depression.

	Liver fat (per 5% increase)	Non‐SLD	SLD	SLD subtypes		
				MASLD	MetALD	ALD
Cross‐sectional analysis (OR [95% CI])						
Cases/total	2849/36 587	1923/26 283	938/10 304	725/7791	151/1838	42/501
Model 1: basic	1.15 (1.11, 1.19)	Reference	1.40 (1.29, 1.52)	1.42 (1.30, 1.56)	1.27 (1.07, 1.51)	1.37 (0.99, 1.89)
Model 2: model 1 + smoking + PA	1.12 (1.08, 1.16)	Reference	1.32 (1.22, 1.44)	1.36 (1.24, 1.49)	1.17 (0.98, 1.40)	1.20 (0.87, 1.66)
Model 3: model 2 + alcohol intake	1.12 (1.09, 1.16)	Reference	1.33 (1.22, 1.45)	1.34 (1.22, 1.48)	1.23 (1.01, 1.49)	1.38 (0.94, 2.02)
Model 4: model 3 + FIB4 score	1.13 (1.09, 1.17)	Reference	1.34 (1.23, 1.46)	1.35 (1.23, 1.49)	1.22 (1.01, 1.48)	1.28 (0.86, 1.89)
Model 5: model 4 + BMI	1.04 (1.01, 1.09)	Reference	1.12 (1.01, 1.23)	1.12 (1.01, 1.25)	1.04 (0.85, 1.27)	1.09 (0.73, 1.62)
Model 6: model 4 + WC	1.04 (1.00, 1.08)	Reference	1.11 (1.01, 1.22)	1.12 (1.01, 1.24)	1.03 (0.84, 1.25)	1.07 (0.72, 1.59)
Model 7: model 4 + WHR	1.09 (1.05, 1.13)	Reference	1.23 (1.12, 1.35)	1.24 (1.12, 1.37)	1.12 (0.92, 1.36)	1.18 (0.79, 1.75)
Model 8: model 4 + WHtR	1.04 (1.00, 1.08)	Reference	1.11 (1.01, 1.22)	1.12 (1.01, 1.24)	1.02 (0.84, 1.25)	1.07 (0.72, 1.60)
Model 9: model 4 + BF%	1.06 (1.02, 1.11)	Reference	1.16 (1.05, 1.28)	1.17 (1.05, 1.30)	1.07 (0.88, 1.31)	1.13 (0.76, 1.69)
Prospective analysis (HR [95% CI])						
Events /total	414/33 726	279/24 360	135/9366	100/7066	25/1687	5/459
Model 1: basic	1.15 (1.06, 1.25)	Reference	1.27 (1.03, 1.56)	1.23 (0.98, 1.55)	1.35 (0.89, 2.04)	1.01 (0.42, 2.46)
Model 2: model 1 + smoking + PA	1.13 (1.04, 1.22)	Reference	1.21 (0.98, 1.49)	1.19 (0.94, 1.51)	1.22 (0.81, 1.85)	0.85 (0.35, 2.08)
Model 3: model 2 + alcohol intake	1.13 (1.04, 1.23)	Reference	1.21 (0.98, 1.50)	1.20 (0.94, 1.52)	1.21 (0.77, 1.89)	0.82 (0.30, 2.26)
Model 4: model 3 + FIB4 score	1.13 (1.03, 1.23)	Reference	1.21 (0.98, 1.51)	1.20 (0.95, 1.53)	1.19 (0.75, 1.88)	0.84 (0.30, 2.34)
Model 5: model 4 + BMI	1.02 (0.92, 1.12)	Reference	0.93 (0.73, 1.18)	0.91 (0.70, 1.19)	0.94 (0.59, 1.49)	0.66 (0.24, 1.84)
Model 6: model 4 + WC	1.02 (0.93, 1.13)	Reference	0.95 (0.75, 1.20)	0.93 (0.72, 1.21)	0.95 (0.59, 1.51)	0.65 (0.23, 1.83)
Model 7: model 4 + WHR	1.10 (1.00, 1.20)	Reference	1.12 (0.89, 1.42)	1.11 (0.86, 1.43)	1.10 (0.69, 1.75)	0.78 (0.28, 2.16)
Model 8: model 4 + WHtR	1.03 (0.94, 1.14)	Reference	0.97 (0.76, 1.23)	0.95 (0.73, 1.24)	0.96 (0.60, 1.53)	0.67 (0.24, 1.86)
Model 9: model 4 + BF%	1.04 (0.95, 1.15)	Reference	1.00 (0.79, 1.26)	0.98 (0.75, 1.27)	1.01 (0.64, 1.62)	0.71 (0.25, 1.98)

*Note*: Basic model: adjusted for sex, age, ethnicity, education and Townsend Deprivation Index.

Abbreviations: ALD, alcohol‐related liver disease; BF%, body fat percentage; BMI, body mass index; CI, confidence interval; HR, hazard ratio; MASLD, metabolic dysfunction‐associated steatotic liver disease; MetALD, metabolic dysfunction and alcohol‐related liver disease; OR, odds ratio; PA, physical activity; SLD, steatotic liver disease; WC, waist circumference; WHR, waist‐to‐hip ratio; WHtR, waist‐to‐height ratio.

For continuous liver fat (for each 5% increases), the association with depression decreased from OR 1.15 (1.11, 1.19) to OR 1.04 (1.01, 1.09), after BMI adjustment. Using WC, WHR, WHtR and BF% as obesity measures also generated similar results. No significant differences were observed between males and females (Table S2).

### 3.2 Prospective associations

After excluding 2849 participants with prevalent depression, 33 762 participants were included in the prospective analysis. Over a median follow‐up of 4.5 years, 414 new cases of depression were recorded.

The results of prospective analyses are shown in the lower panel of Table 2. In the basic model, SLD was associated with a 27% higher risk of depression (HR [95% CI]: 1.27 [1.03, 1.56]). Additionally adjusting for smoking, physical activity and alcohol intake slightly weakened the associations, with a wider confidence interval, but the point estimate remained similar. However, further adjustment for BMI reduced the association to 0.93 (0.73, 1.18). Similar results were observed for SLD subtypes, with no significant associations after BMI adjustment. For liver fat, the association weakened from 1.15 (1.06, 1.25) to 1.02 (0.92, 1.12) after BMI adjustment, indicating a null association. Using WC, WHR, WHtR and BF% as obesity measures also generated similar results. No sex difference was observed (Table S3).

**FIGURE 2:**
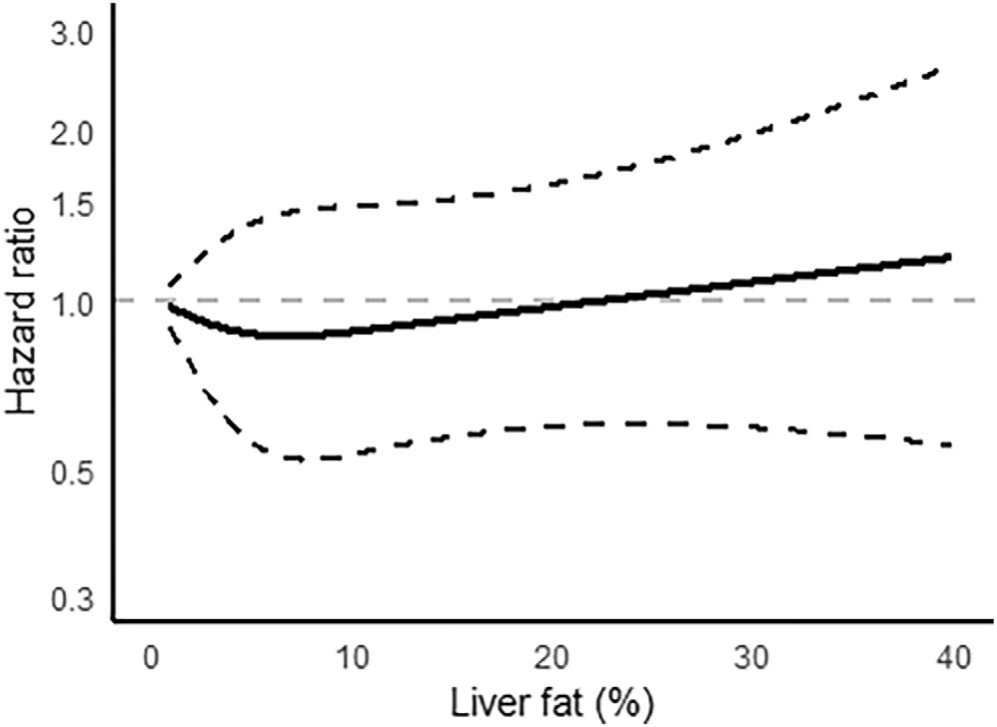
Non‐linear association between liver fat and incidence of depression. Liver fat was fitted as a restricted cubic spline function with 4 knots. Results from a Cox model adjusted for age, sex, ethnicity, education, Townsend Deprivation Index, smoking, physical activity, alcohol intake, FIB4 score and body mass index.

Non‐linear modelling of liver fat similarly confirmed a null association with depression after BMI adjustment (Figure 2).

In sensitivity analysis excluding the first two years of follow‐up, we also observed that the associations were partially attenuated compared with the primary analysis and exhibited similarly null associations after adjustment for BMI (Table S4). Examining the association between liver fat severity and prevalence and incidence of depression also showed similar results (Table S5).

Subgroup analysis by age group, Townsend Deprivation Index (fifths), education, smoking, physical activity and SLD subtypes consistently showed null associations after adjusting for BMI, except in individuals with low physical activity, where each 5% increase in liver fat was associated with a 29% higher risk of depression (1.29 [1.07, 1.54]), although the subgroup difference did not reach the statistical significance level (Table S6).

## 4 DISCUSSION

In this large cohort of 36 587 participants with MRI‐derived liver fat measurement, we found that participants with SLD or higher liver fat percentage exhibited higher prevalence and incidence of depression. However, these associations were partially attenuated after adjusting for socioeconomic status and lifestyle factors, and were substantially attenuated after accounting for obesity measures, suggesting that obesity was an important confounder in previously observed associations. Sensitivity analyses and subgroup analyses confirmed these findings.

Previous systematic reviews of observational studies have generally reported positive associations between MASLD and depression.^14^, ^18^, ^19^ For example, Gu et al.^18^ analysed seven cross‐sectional studies and reported that MASLD was associated with a 13% higher risk of depression, although six of these studies were cross‐sectional, limiting causal inference. Xiao et al.^19^ reviewed four cross‐sectional studies and found that both MASLD and MASLD severity were positively associated with depression. Additionally, Ng et al.^27^ observed a positive association between fatty liver index (a biomarker surrogate for liver steatosis) and depression prevalence in a cross‐sectional study of the US population. However, cross‐sectional studies are prone to residual confounding and reverse causation, which undermines their validity.

Our findings of null prospective associations between liver fat and depression align with Brodosi et al.,^21^ which similarly reported that the initially observed association between MASLD and depression prevalence became non‐significant after adjusting for socioeconomic status (age, sex education) and BMI. Interestingly, their unadjusted analysis observed an inverse association, contradicting most prior evidence.^18^, ^19^, ^20^, ^27^ They suggested that older age and male sex in the MASLD population might contribute to a lower depression prevalence, as both factors have been linked to lower prevalence of depression.^21^ Furthermore, compared with Brodosi et al., our study had several advantages, including providing prospective evidence, a larger cohort (36 587 vs. 286) and clinically confirmed diagnosis of depression. Additionally, our findings aligned with recent Mendelian randomization studies, which did not support a causal relationship between MASLD and depression.^22^

By contrast, Åström et al.^20^ and Labenz et al.^28^ reported positive prospective associations between MASLD and depression. The two studies had similar study designs, which, however, may have contributed to potential misclassification bias. Both studies used clinically diagnosed MASLD, identified matched comparators for MASLD cases based on a series of variables, and considered clinically diagnosed obesity in their analysis. Therefore, using only diagnostic codes for MASLD and obesity likely underestimates their prevalences by 42% and 45%, respectively.^29^ As a result, individuals with mild MASLD were likely misclassified as non‐MASLD, and those with obesity but no clinical diagnosis were classified as non‐obese, potentially leading to an underestimation of both MASLD and obesity prevalence. For example, in a study by Åström et al., only 13.0% of people with MASLD had obesity, which was substantially lower than the prevalence in our study and other large cohorts.^30^ This discrepancy suggests that their MASLD sample may have been skewed toward individuals with more metabolic dysfunction and comorbidities, and that the adjustment for clinical obesity was not as efficient as adjustment for BMI or other obesity measures, possibly explaining their observed positive association.

Prior studies have suggested that depression itself increases the risk of MASLD, rather than the reverse. This is supported consistently by observational studies^31^, ^32^, ^33^ and Mendelian randomization analyses.^22^, ^32^ Proposed mechanisms include the effects of antidepressants and antipsychotics, modifying body weight, energy metabolism and neurotransmitter pathways, including dopaminergic, serotonergic, histaminergic and muscarinic signalling.^34^ Additionally, genetic, lifestyle and environmental factors shared between MASLD and depression—including, diet, physical activity and chronic stress—may contribute to this relationship, via insulin resistance, dysregulation of inflammation, oxidative stress, mitochondrial dysfunction and gut microbiota alterations.^34^, ^35^, ^36^, ^37^ Moreover, liver fat accumulation has been associated with widespread metabolic changes, particularly alterations in low‐density lipoproteins, glycolysis and inflammatory markers, amino acids and fatty acids, which have widespread health effects, including mental health.^38^

Reverse causation is an important consideration when interpreting our findings, particularly in the cross‐sectional analysis. In this context, reverse causation refers to the possibility that depression may lead to increased liver fat accumulation—not liver fat increasing the risk of depression. This is plausible given that depression can influence risk factors for hepatic steatosis. However, our prospective analysis, in which liver fat was assessed at baseline and incident depression was ascertained during follow‐up, helps establish a clearer temporal sequence. To further minimize the potential for reverse causation, we conducted a sensitivity analysis excluding the first two years of follow‐up, and the results remained consistent with the primary prospective analysis.

This study has several strengths, including the use of a large, well‐characterized cohort with detailed phenotyping of liver fat, metabolic risk factors and depression. The availability of longitudinal data allowed us to examine both cross‐sectional and prospective associations.

However, some limitations should be acknowledged. First, UK Biobank participants are known to be more socioeconomically advantaged and healthier than the general UK population,^39^ which may limit the generalizability of our findings, particularly in underrepresented and high‐risk populations. Additionally, the average age of participants in our study was 64.5 years, which may restrict applicability to populations of different age groups. Second, the biochemistry data were measured at the baseline visit, while the other data were measured at the imaging visit, which was, on average, 9 years after the baseline visit. Third, we were unable to examine the associations with depression subtypes (e.g., atypical vs. melancholic) due to a lack of such data in UK Biobank. Fourth, the median follow‐up duration of 4.5 years was relatively short, and longer follow‐up may provide more conclusive evidence. Fifth, depression diagnoses were ascertained using hospital records supplemented by self‐reported data, which may have preferentially captured more severe cases and missed milder cases that are managed at primary care settings. Finally, although we adjusted for a range of covariates, residual confounding cannot be ruled out, as is common in observational studies.

Given our findings, the observed association between SLD and depression in cross‐sectional analyses is likely due to both reverse causation and confounding of obesity, whereas the prospective association is primarily driven by obesity. The association between obesity and depression is well established.^40^ This aligns with our results showing that, in cross‐sectional analyses, a weak association remained after obesity adjustment (suggesting reverse causation), whereas in prospective analyses, the association disappeared entirely. Future studies are warranted to further confirm this.

Our findings have important clinical and public health implications. Given that obesity appears to explain much of the association between liver fat and depression, targeting obesity through weight management interventions may be a more effective strategy for reducing depression risk in individuals with SLD.

## 5 CONCLUSION

In summary, we found that SLD was associated with a higher prevalence of depression, but this association was largely explained by obesity measures. In the prospective analysis, SLD did not predict future depression risk after adjusting for obesity, suggesting that obesity may be the key factor confounding this relationship. These findings emphasize the importance of weight management in individuals with SLD, not only for metabolic health but also for mental well‐being. Future studies should further explore the role of obesity (including the impact of weight reduction on depression), lifestyle interventions and potential biological mechanisms linking liver fat and mental health outcomes.

## AUTHOR CONTRIBUTIONS

QF conceived the research idea, conducted data analysis and drafted the manuscript. All authors interpreted results, critically reviewed and revised the manuscript.

## FUNDING INFORMATION

This research/study/project was funded/supported by the NIHR Imperial Biomedical Research Centre (BRC) (NIHR203323). The views expressed are those of the author(s) and not necessarily those of the NIHR or the Department of Health and Social Care. The Division of Digestive Diseases at Imperial College London receives financial support from the National Institute of Health Research (NIHR) Imperial Biomedical Research Centre (BRC) based at Imperial College London and Imperial College Healthcare NHS Trust.

## CONFLICT OF INTEREST STATEMENT

CI has conducted consultancy work for Novo Nordisk outside the submitted work.

## PEER REVIEW

The peer review history for this article is available at https://www.webofscience.com/api/gateway/wos/peer-review/10.1111/dom.16562.

## ETHICS STATEMENT

The authors assert that all procedures contributing to this work comply with the ethical standards of the relevant national and institutional committees on human experimentation and with the Helsinki Declaration of 1975, as revised in 2000. Our analyses were based on UK Biobank data, under the approved application #74018. UK Biobank was approved by the North West Multi‐centre Research Ethics Committee as a Research Tissue Bank approval. No extra ethical approval is required for this specific study.

## INFORMED CONSENT

All participants have provided informed consent to participate in UK Biobank. The authors affirm that human research participants provided informed consent for publication of this manuscript.

## Supporting information


**Table S1.** Baseline characteristics of participants with steatotic liver disease, stratified by steatotic liver disease subtypes.
**Table S2.** Cross‐sectional associations between liver fat, SLD, SLD subtypes and prevalence of depression (expanded).
**Table S3.** Prospective associations between liver fat, SLD, SLD subtypes and incidence of depression (expanded).
**Table S4.** Sensitivity analysis for prospective associations between liver fat, SLD, SLD subtypes and incidence of depression, excluding the first 2 years of follow‐up.
**Table S5.** Cross‐sectional and prospective associations between liver fat severity and depression.
**Table S6.** Subgroup analysis for the associations between liver fat (per 5%) and incidence of depression.

## Data Availability

UK Biobank data are available to registered researchers at https://www.ukbiobank.ac.uk/.
