# Supplementary material for: Obesity drives the link between liver fat and depression: Cross‐sectional and prospective investigations
Source: Diabetes Obes Metab. 2025 Jun 24;27(9):5127–34. doi: 10.1111/dom.16562 (PMC12326890; doi:10.1111/dom.16562)
Supplement: Supplementary file 1 — Table S1. Baseline characteristics of participants with steatotic liver disease, stratified by steatotic liver disease subtypes. Table S2. Cross‐sectional associations between liver fat, SLD, SLD subtypes and prevalence of depression (expanded). Table S3. Prospective associations between liver fat, SLD, SLD subtypes and incidence of depression (expanded). Table S4. Sensitivity analysis for prospective associations between liver fat, SLD, SLD subtypes and incidence of depression, excluding the first 2 years of follow‐up. Table S5. Cross‐sectional and prospective associations between liver fat severity and depression. Table S6. Subgroup analysis for the associations between liver fat (per 5%) and incidence of depression. [file DOM-27-5127-s001.docx]

Obesity drives the link between liver fat and depression: cross-sectional and prospective investigations

Supplementary material

### Supplementary table 1: Baseline characteristics of participants with steatotic liver disease, stratified by steatotic liver disease subtypes

|  | MASLD | MetALD | ALD |
| --- | --- | --- | --- |
|  | n = 7791 | n = 1838 | n = 501 |
| Sex, female | 3270 (42.0%) | 600 (33.6%) | 407 (18.8%) |
| Age, years | 64.9 (7.5) | 64.2 (7.1) | 63.5 (7.4) |
| Townsend deprivation index |  |  |  |
| 1st fifth (least deprived) | 1481 (19%) | 360 (19.6%) | 91 (18.2%) |
| 5th fifth (most deprived) | 1665 (21.4%) | 382 (20.8%) | 110 (22.0%) |
| Education, Higher education | 3295 (42.3%) | 773 (42.1%) | 210 (41.9%) |
| Smoking, never | 4887 (62.7%) | 810 (44.1%) | 190 (37.9%) |
| Alcohol intake, g/day | 9.1 (8.4) | 37.9 (10.1) | 78.4 (21.8) |
| Physical activity, high | 2500 (32.1%) | 664 (36.1%) | 162 (32.3%) |
| Liver fat, % | 10.7 (6.0) | 10.8 (6.2) | 12.4 (7.4) |
|  | 8.6 (6.3, 13.2) | 8.6 (6.4, 13.2) | 9.7 (6.8, 16.1) |
| Body mass index, kg/m^2^ | 29.8 (4.4) | 29.3 (4.1) | 29.3 (4.2) |
| Waist circumference, cm | 97.6 (11.3) | 97.7 (10.8) | 99.6 (11.2) |
| Waist-to-hip ratio, % | 92.5 (7.9) | 93.4 (7.4) | 95.3 (7.0) |
| Waist-to-height ratio, % | 57.5 (6.4) | 57.0 (6.2) | 57.3 (6.2) |
| Body fat, % | 34.1 (7.9) | 32.5 (7.4) | 31.1 (6.6) |
| Systolic blood pressure, mmHg | 143.8 (17.3) | 145.3 (17.2) | 148.2 (18.7) |
| Diastolic blood pressure, mmHg | 81.7 (9.7) | 83.0 (9.5) | 84.3 (9.9) |
| Hypertension | 7140 (91.6%) | 1710 (93.0%) | 476 (95.0%) |
| Obesity | 7156 (91.8%) | 1687 (91.8%) | 444 (88.6%) |
| Diabetes | 1861 (23.9%) | 317 (17.2%) | 78 (15.6%) |
| High triglycerides | 4660 (59.8%) | 984 (53.5%) | 285 (56.9%) |
| Low HDL-cholesterol | 3399 (43.6%) | 549 (29.9%) | 116 (23.2%) |

MASLD: metabolic dysfunction associated steatotic liver disease. MetALD: metabolic dysfunction and alcohol related liver disease. ALD: alcohol related liver disease. HDL: high density lipoprotein. NA: not applicable.

### Supplementary table 2: Cross-sectional associations between liver fat, SLD, SLD subtypes and prevalence of depression (expanded).

|  |  |  |  | SLD subtypes |  |  |  |
| --- | --- | --- | --- | --- | --- | --- | --- |
|  | Liver fat  (per 5% increase) | Non SLD | SLD | MASLD | MetALD | ALD | Others |
| **Females + males** |  |  |  |  |  |  |  |
| Cases/total | 2849 / 36587 | 1923 / 26283 | 938 / 10304 | 725 / 7791 | 151 / 1838 | 42 / 501 | 20 / 174 |
| Model 1: basic | 1.15 (1.11, 1.19) | Reference | 1.40 (1.29, 1.52) | 1.42 (1.30, 1.56) | 1.27 (1.07, 1.51) | 1.37 (0.99, 1.89) | 1.78 (1.11, 2.85) |
| Model 2: model 1 + smoking + PA | 1.12 (1.08, 1.16) | Reference | 1.32 (1.22, 1.44) | 1.36 (1.24, 1.49) | 1.17 (0.98, 1.40) | 1.20 (0.87, 1.66) | 1.65 (1.03, 2.65) |
| Model 3: model 2 + alcohol intake | 1.12 (1.09, 1.16) | Reference | 1.33 (1.22, 1.45) | 1.34 (1.22, 1.48) | 1.23 (1.01, 1.49) | 1.38 (0.94, 2.02) | 1.65 (1.03, 2.66) |
| Model 4: model 3 + FIB4 score | 1.13 (1.09, 1.17) | Reference | 1.34 (1.23, 1.46) | 1.35 (1.23, 1.49) | 1.22 (1.01, 1.48) | 1.28 (0.86, 1.89) | 1.72 (1.07, 2.76) |
| Model 5: model 4 + BMI | 1.04 (1.01, 1.09) | Reference | 1.12 (1.01, 1.23) | 1.12 (1.01, 1.25) | 1.04 (0.85, 1.27) | 1.09 (0.73, 1.62) | 1.57 (0.97, 2.53) |
| Model 6: model 4 + WC | 1.04 (1.00, 1.08) | Reference | 1.11 (1.00, 1.22) | 1.12 (1.00, 1.24) | 1.03 (0.84, 1.25) | 1.07 (0.72, 1.59) | 1.48 (0.92, 2.39) |
| Model 7: model 4 + WHR | 1.09 (1.05, 1.13) | Reference | 1.23 (1.12, 1.35) | 1.24 (1.12, 1.37) | 1.12 (0.92, 1.36) | 1.18 (0.79, 1.75) | 1.59 (0.99, 2.56) |
| Model 8: model 4 + WHtR | 1.04 (1.00, 1.08) | Reference | 1.11 (1.01, 1.22) | 1.12 (1.01, 1.24) | 1.02 (0.84, 1.25) | 1.07 (0.72, 1.60) | 1.52 (0.94, 2.45) |
| Model 9: model 4 + BF% | 1.06 (1.02, 1.11) | Reference | 1.16 (1.05, 1.28) | 1.17 (1.05, 1.30) | 1.07 (0.88, 1.31) | 1.13 (0.76, 1.69) | 1.60 (0.99, 2.57) |
| **Females** |  |  |  |  |  |  |  |
| Cases/total | 1780 / 18794 | 1308 / 14766 | 481 / 4028 | 381 / 3270 | 76 / 600 | 17 / 94 | 7 / 64 |
| Model 1: basic | 1.16 (1.11, 1.21) | Reference | 1.43 (1.28, 1.60) | 1.40 (1.24, 1.59) | 1.49 (1.17, 1.92) | 2.15 (1.26, 3.65) | 1.22 (0.56, 2.70) |
| Model 2: model 1 + smoking + PA | 1.13 (1.08, 1.18) | Reference | 1.36 (1.22, 1.52) | 1.34 (1.19, 1.52) | 1.39 (1.08, 1.78) | 1.92 (1.13, 3.27) | 1.10 (0.50, 2.44) |
| Model 3: model 2 + alcohol intake | 1.13 (1.08, 1.18) | Reference | 1.36 (1.21, 1.52) | 1.35 (1.19, 1.53) | 1.38 (1.06, 1.80) | 1.88 (1.03, 3.43) | 1.10 (0.50, 2.44) |
| Model 4: model 3 + FIB4 score | 1.14 (1.09, 1.19) | Reference | 1.38 (1.23, 1.55) | 1.38 (1.21, 1.56) | 1.36 (1.03, 1.79) | 1.90 (1.04, 3.48) | 1.16 (0.52, 2.57) |
| Model 5: model 4 + BMI | 1.04 (0.99, 1.10) | Reference | 1.12 (0.98, 1.27) | 1.10 (0.96, 1.27) | 1.12 (0.85, 1.48) | 1.61 (0.88, 2.96) | 1.06 (0.48, 2.36) |
| Model 6: model 4 + WC | 1.04 (0.98, 1.10) | Reference | 1.11 (0.98, 1.27) | 1.10 (0.96, 1.27) | 1.10 (0.83, 1.46) | 1.56 (0.85, 2.86) | 0.98 (0.44, 2.19) |
| Model 7: model 4 + WHR | 1.10 (1.05, 1.16) | Reference | 1.28 (1.13, 1.45) | 1.27 (1.11, 1.46) | 1.25 (0.95, 1.65) | 1.76 (0.96, 3.23) | 1.08 (0.49, 2.39) |
| Model 8: model 4 + WHtR | 1.04 (0.99, 1.10) | Reference | 1.12 (0.98, 1.28) | 1.11 (0.96, 1.28) | 1.11 (0.83, 1.47) | 1.58 (0.86, 2.89) | 1.03 (0.46, 2.29) |
| Model 9: model 4 + BF% | 1.07 (1.01, 1.12) | Reference | 1.19 (1.05, 1.35) | 1.19 (1.03, 1.36) | 1.15 (0.87, 1.53) | 1.72 (0.93, 3.15) | 1.11 (0.50, 2.48) |
| **Males** |  |  |  |  |  |  |  |
| Cases/total | 1069 / 17793 | 615 / 11517 | 457 / 6276 | 344 / 4521 | 75 / 1238 | 25 / 407 | 13 / 110 |
| Model 1: basic | 1.14 (1.09, 1.20) | Reference | 1.36 (1.20, 1.55) | 1.44 (1.25, 1.65) | 1.10 (0.86, 1.41) | 1.10 (0.73, 1.66) | 2.33 (1.29, 4.19) |
| Model 2: model 1 + smoking + PA | 1.11 (1.05, 1.17) | Reference | 1.28 (1.12, 1.45) | 1.37 (1.19, 1.57) | 1.00 (0.78, 1.29) | 0.95 (0.63, 1.44) | 2.20 (1.22, 3.97) |
| Model 3: model 2 + alcohol intake | 1.12 (1.06, 1.18) | Reference | 1.31 (1.15, 1.49) | 1.34 (1.16, 1.54) | 1.10 (0.83, 1.44) | 1.21 (0.73, 1.99) | 2.21 (1.23, 3.98) |
| Model 4: model 3 + FIB4 score | 1.11 (1.05, 1.17) | Reference | 1.29 (1.13, 1.47) | 1.32 (1.15, 1.53) | 1.10 (0.83, 1.44) | 1.04 (0.62, 1.76) | 2.25 (1.25, 4.06) |
| Model 5: model 4 + BMI | 1.04 (0.98, 1.11) | Reference | 1.11 (0.96, 1.29) | 1.14 (0.97, 1.33) | 0.96 (0.73, 1.27) | 0.91 (0.53, 1.54) | 2.06 (1.14, 3.72) |
| Model 6: model 4 + WC | 1.04 (0.98, 1.11) | Reference | 1.10 (0.96, 1.28) | 1.13 (0.97, 1.32) | 0.95 (0.72, 1.26) | 0.90 (0.53, 1.53) | 1.98 (1.09, 3.58) |
| Model 7: model 4 + WHR | 1.07 (1.01, 1.13) | Reference | 1.17 (1.02, 1.34) | 1.20 (1.03, 1.40) | 1.00 (0.76, 1.32) | 0.95 (0.56, 1.61) | 2.07 (1.15, 3.75) |
| Model 8: model 4 + WHtR | 1.04 (0.98, 1.10) | Reference | 1.10 (0.95, 1.27) | 1.12 (0.96, 1.31) | 0.94 (0.71, 1.25) | 0.89 (0.52, 1.50) | 1.99 (1.10, 3.60) |
| Model 9: model 4 + BF% | 1.06 (1.00, 1.12) | Reference | 1.12 (0.97, 1.30) | 1.14 (0.97, 1.34) | 0.99 (0.75, 1.31) | 0.92 (0.53, 1.57) | 2.02 (1.12, 3.67) |

Basic Model: adjusted for sex, age, ethnicity, education and Townsend Deprivation Index. SLD: steatotic liver disease. MASLD: metabolic dysfunction associated steatotic liver disease. MetALD: metabolic dysfunction and alcohol related liver disease. ALD: alcohol related liver disease. PA: physical activity. BMI: body mass index. WC: waist circumference. WHR: waist-to-hip ratio. WHtR: waist-to-height ratio. BF%: body fat percentage.

### Supplementary table 3: Prospective associations between liver fat, SLD, SLD subtypes and incidence of depression (expanded).

|  |  |  |  | SLD subtypes |  |  |  |
| --- | --- | --- | --- | --- | --- | --- | --- |
|  | Liver fat  (per 5% increase) | Non SLD | SLD | MASLD | MetALD | ALD | Others |
| Females + males |  |  |  |  |  |  |  |
| Cases/total | 414 / 33726 | 279 / 24360 | 135 / 9366 | 100 / 7066 | 25 / 1687 | 5 / 459 | 5 / 154 |
| Model 1: basic | 1.15 (1.06, 1.25) | Reference | 1.27 (1.03, 1.56) | 1.23 (0.98, 1.55) | 1.35 (0.89, 2.04) | 1.01 (0.42, 2.46) | 3.06 (1.26, 7.41) |
| Model 2: model 1 + smoking + PA | 1.13 (1.04, 1.22) | Reference | 1.21 (0.98, 1.49) | 1.19 (0.94, 1.51) | 1.22 (0.81, 1.85) | 0.85 (0.35, 2.08) | 2.78 (1.15, 6.75) |
| Model 3: model 2 + alcohol intake | 1.13 (1.04, 1.23) | Reference | 1.21 (0.98, 1.50) | 1.20 (0.94, 1.52) | 1.21 (0.77, 1.89) | 0.82 (0.30, 2.26) | 2.78 (1.15, 6.75) |
| Model 4: model 3 + FIB4 score | 1.13 (1.03, 1.23) | Reference | 1.21 (0.98, 1.51) | 1.20 (0.95, 1.53) | 1.19 (0.75, 1.88) | 0.84 (0.30, 2.34) | 2.92 (1.20, 7.08) |
| Model 5: model 4 + BMI | 1.02 (0.92, 1.12) | Reference | 0.93 (0.73, 1.18) | 0.91 (0.70, 1.19) | 0.94 (0.59, 1.49) | 0.66 (0.24, 1.84) | 2.45 (1.01, 5.98) |
| Model 6: model 4 + WC | 1.02 (0.93, 1.13) | Reference | 0.95 (0.75, 1.20) | 0.93 (0.72, 1.21) | 0.95 (0.59, 1.51) | 0.65 (0.23, 1.83) | 2.36 (0.97, 5.76) |
| Model 7: model 4 + WHR | 1.10 (1.00, 1.20) | Reference | 1.12 (0.89, 1.42) | 1.11 (0.86, 1.43) | 1.10 (0.69, 1.75) | 0.78 (0.28, 2.16) | 2.73 (1.12, 6.64) |
| Model 8: model 4 + WHtR | 1.03 (0.94, 1.14) | Reference | 0.97 (0.76, 1.23) | 0.95 (0.73, 1.24) | 0.96 (0.60, 1.53) | 0.67 (0.24, 1.86) | 2.51 (1.03, 6.11) |
| Model 9: model 4 + BF% | 1.04 (0.95, 1.15) | Reference | 1.00 (0.79, 1.26) | 0.98 (0.75, 1.27) | 1.01 (0.64, 1.62) | 0.71 (0.25, 1.98) | 2.53 (1.04, 6.16) |
| Females |  |  |  |  |  |  |  |
| Cases/total | 237 / 17005 | 170 / 13458 | 67 / 3547 | 55 / 2889 | 8 / 524 | 2 / 77 | 2 / 57 |
| Model 1: basic | 1.20 (1.08, 1.33) | Reference | 1.47 (1.11, 1.96) | 1.48 (1.09, 2.01) | 1.20 (0.59, 2.44) | 1.99 (0.49, 8.02) | 2.65 (0.66, 10.71) |
| Model 2: model 1 + smoking + PA | 1.17 (1.05, 1.30) | Reference | 1.37 (1.03, 1.82) | 1.40 (1.02, 1.90) | 1.08 (0.53, 2.20) | 1.67 (0.41, 6.75) | 2.25 (0.56, 9.13) |
| Model 3: model 2 + alcohol intake | 1.17 (1.05, 1.30) | Reference | 1.38 (1.03, 1.84) | 1.34 (0.98, 1.83) | 1.33 (0.62, 2.87) | 3.01 (0.61, 14.84) | 2.24 (0.55, 9.09) |
| Model 4: model 3 + FIB4 score | 1.17 (1.05, 1.31) | Reference | 1.39 (1.04, 1.86) | 1.37 (1.00, 1.88) | 1.20 (0.53, 2.72) | 3.11 (0.63, 15.31) | 2.33 (0.57, 9.47) |
| Model 5: model 4 + BMI | 1.05 (0.93, 1.20) | Reference | 1.04 (0.75, 1.44) | 1.01 (0.71, 1.44) | 0.92 (0.40, 2.10) | 2.46 (0.49, 12.19) | 1.94 (0.48, 7.94) |
| Model 6: model 4 + WC | 1.06 (0.93, 1.21) | Reference | 1.06 (0.76, 1.48) | 1.05 (0.73, 1.49) | 0.93 (0.41, 2.13) | 2.35 (0.47, 11.74) | 1.86 (0.46, 7.63) |
| Model 7: model 4 + WHR | 1.15 (1.02, 1.30) | Reference | 1.32 (0.96, 1.80) | 1.30 (0.93, 1.81) | 1.13 (0.50, 2.58) | 2.93 (0.59, 14.52) | 2.21 (0.54, 9.02) |
| Model 8: model 4 + WHtR | 1.07 (0.94, 1.22) | Reference | 1.07 (0.77, 1.49) | 1.05 (0.74, 1.50) | 0.93 (0.40, 2.12) | 2.40 (0.48, 11.99) | 2.00 (0.49, 8.16) |
| Model 9: model 4 + BF% | 1.09 (0.96, 1.23) | Reference | 1.13 (0.82, 1.56) | 1.11 (0.78, 1.56) | 1.02 (0.45, 2.34) | 2.76 (0.55, 13.78) | 2.04 (0.50, 8.33) |
| Males |  |  |  |  |  |  |  |
| Cases/total | 177 / 16721 | 109 / 10902 | 68 / 5819 | 45 / 4177 | 17 / 1163 | 3 / 382 | 3 / 97 |
| Model 1: basic | 1.10 (0.97, 1.25) | Reference | 1.11 (0.82, 1.50) | 1.02 (0.72, 1.45) | 1.39 (0.83, 2.32) | 0.71 (0.22, 2.24) | 3.29 (1.05, 10.38) |
| Model 2: model 1 + smoking + PA | 1.09 (0.96, 1.23) | Reference | 1.08 (0.79, 1.47) | 1.02 (0.72, 1.45) | 1.27 (0.76, 2.12) | 0.61 (0.19, 1.94) | 3.28 (1.04, 10.34) |
| Model 3: model 2 + alcohol intake | 1.08 (0.95, 1.23) | Reference | 1.07 (0.78, 1.45) | 1.06 (0.74, 1.52) | 1.13 (0.65, 1.96) | 0.44 (0.12, 1.61) | 3.27 (1.04, 10.30) |
| Model 4: model 3 + FIB4 score | 1.08 (0.94, 1.23) | Reference | 1.06 (0.77, 1.45) | 1.04 (0.72, 1.50) | 1.16 (0.67, 2.02) | 0.46 (0.12, 1.67) | 3.38 (1.07, 10.69) |
| Model 5: model 4 + BMI | 1.00 (0.86, 1.16) | Reference | 0.87 (0.62, 1.24) | 0.85 (0.57, 1.26) | 0.96 (0.54, 1.71) | 0.37 (0.10, 1.38) | 3.00 (0.94, 9.51) |
| Model 6: model 4 + WC | 0.99 (0.85, 1.15) | Reference | 0.87 (0.61, 1.23) | 0.85 (0.57, 1.26) | 0.96 (0.54, 1.71) | 0.37 (0.10, 1.37) | 2.82 (0.89, 8.99) |
| Model 7: model 4 + WHR | 1.04 (0.90, 1.20) | Reference | 0.96 (0.69, 1.35) | 0.94 (0.64, 1.38) | 1.06 (0.60, 1.87) | 0.41 (0.11, 1.51) | 3.11 (0.98, 9.85) |
| Model 8: model 4 + WHtR | 1.01 (0.87, 1.17) | Reference | 0.90 (0.64, 1.28) | 0.88 (0.59, 1.30) | 0.99 (0.56, 1.76) | 0.38 (0.10, 1.41) | 2.97 (0.94, 9.45) |
| Model 9: model 4 + BF% | 1.01 (0.87, 1.18) | Reference | 0.92 (0.65, 1.30) | 0.89 (0.60, 1.32) | 1.04 (0.59, 1.83) | 0.39 (0.11, 1.45) | 3.02 (0.95, 9.61) |

Basic Model: adjusted for sex, age, ethnicity, education and Townsend Deprivation Index. SLD: steatotic liver disease. MASLD: metabolic dysfunction associated steatotic liver disease. MetALD: metabolic dysfunction and alcohol related liver disease. ALD: alcohol related liver disease. PA: physical activity. BMI: body mass index. WC: waist circumference. WHR: waist-to-hip ratio. WHtR: waist-to-height ratio. BF%: body fat percentage.

### Supplementary table 4: Sensitivity analysis for prospective associations between liver fat, SLD, SLD subtypes and incidence of depression, excluding the first two years of follow-up.

|  |  |  |  | SLD subtypes |  |  |  |
| --- | --- | --- | --- | --- | --- | --- | --- |
|  | Liver fat (per 5%) | non-SLD | SLD | MASLD | MetALD | ALD | Others |
| Females + males |  |  |  |  |  |  |  |
| Cases/total | 266 / 33578 | 181 / 24262 | 85 / 9316 | 67 / 7033 | 12 / 1674 | 3 / 457 | 3 / 152 |
| Model 1: basic | 1.11 (0.99, 1.23) | Reference | 1.21 (0.93, 1.57) | 1.25 (0.94, 1.66) | 0.97 (0.54, 1.74) | 0.90 (0.29, 2.82) | 2.76 (0.88, 8.65) |
| Model 2: model 1 + smoking + PA | 1.09 (0.98, 1.21) | Reference | 1.17 (0.90, 1.52) | 1.24 (0.93, 1.66) | 0.87 (0.48, 1.56) | 0.75 (0.24, 2.37) | 2.51 (0.80, 7.87) |
| Model 3: model 2 + alcohol intake | 1.09 (0.98, 1.22) | Reference | 1.18 (0.90, 1.54) | 1.25 (0.94, 1.68) | 0.84 (0.45, 1.57) | 0.69 (0.19, 2.52) | 2.51 (0.80, 7.87) |
| Model 4: model 3 + FIB4 score | 1.09 (0.97, 1.21) | Reference | 1.17 (0.89, 1.54) | 1.25 (0.93, 1.68) | 0.80 (0.42, 1.53) | 0.71 (0.19, 2.60) | 2.64 (0.84, 8.28) |
| Model 5: model 4 + BMI | 0.97 (0.85, 1.10) | Reference | 0.89 (0.66, 1.21) | 0.95 (0.68, 1.31) | 0.62 (0.32, 1.21) | 0.56 (0.15, 2.05) | 2.26 (0.72, 7.13) |
| Model 6: model 4 + WC | 0.97 (0.85, 1.11) | Reference | 0.90 (0.66, 1.22) | 0.96 (0.69, 1.32) | 0.62 (0.32, 1.21) | 0.55 (0.15, 2.02) | 2.13 (0.68, 6.74) |
| Model 7: model 4 + WHR | 1.07 (0.95, 1.20) | Reference | 1.12 (0.84, 1.50) | 1.19 (0.87, 1.64) | 0.76 (0.39, 1.47) | 0.68 (0.18, 2.49) | 2.53 (0.80, 7.99) |
| Model 8: model 4 + WHtR | 0.98 (0.86, 1.12) | Reference | 0.93 (0.68, 1.25) | 0.98 (0.71, 1.36) | 0.64 (0.33, 1.23) | 0.56 (0.15, 2.08) | 2.29 (0.73, 7.22) |
| Model 9: model 4 + BF% | 0.98 (0.86, 1.12) | Reference | 0.94 (0.70, 1.26) | 0.99 (0.72, 1.36) | 0.68 (0.35, 1.31) | 0.62 (0.17, 2.29) | 2.30 (0.73, 7.24) |
| Females |  |  |  |  |  |  |  |
| Cases/total | 150 / 16918 | 107 / 13395 | 43 / 3523 | 36 / 2870 | 5 / 521 | 1 / 76 | 1 / 56 |
| Model 1: basic | 1.18 (1.03, 1.35) | Reference | 1.51 (1.05, 2.15) | 1.55 (1.06, 2.27) | 1.19 (0.48, 2.91) | 1.57 (0.22, 11.25) | 2.15 (0.30, 15.48) |
| Model 2: model 1 + smoking + PA | 1.16 (1.01, 1.33) | Reference | 1.44 (1.01, 2.06) | 1.51 (1.03, 2.22) | 1.06 (0.43, 2.60) | 1.32 (0.18, 9.49) | 1.84 (0.26, 13.29) |
| Model 3: model 2 + alcohol intake | 1.17 (1.02, 1.34) | Reference | 1.45 (1.01, 2.08) | 1.43 (0.97, 2.11) | 1.41 (0.53, 3.73) | 2.95 (0.33, 26.54) | 1.84 (0.26, 13.27) |
| Model 4: model 3 + FIB4 score | 1.17 (1.01, 1.34) | Reference | 1.47 (1.02, 2.12) | 1.48 (1.00, 2.19) | 1.16 (0.40, 3.38) | 2.97 (0.33, 26.74) | 1.97 (0.27, 14.18) |
| Model 5: model 4 + BMI | 1.06 (0.90, 1.25) | Reference | 1.15 (0.76, 1.74) | 1.15 (0.74, 1.78) | 0.92 (0.31, 2.72) | 2.46 (0.27, 22.30) | 1.73 (0.24, 12.57) |
| Model 6: model 4 + WC | 1.06 (0.90, 1.26) | Reference | 1.16 (0.77, 1.77) | 1.17 (0.75, 1.82) | 0.93 (0.31, 2.74) | 2.38 (0.26, 21.74) | 1.64 (0.23, 11.94) |
| Model 7: model 4 + WHR | 1.16 (1.00, 1.35) | Reference | 1.46 (0.98, 2.16) | 1.47 (0.97, 2.23) | 1.15 (0.39, 3.38) | 2.95 (0.33, 26.62) | 1.95 (0.27, 14.12) |
| Model 8: model 4 + WHtR | 1.07 (0.90, 1.26) | Reference | 1.17 (0.77, 1.78) | 1.18 (0.76, 1.83) | 0.92 (0.31, 2.74) | 2.42 (0.26, 22.08) | 1.77 (0.24, 12.81) |
| Model 9: model 4 + BF% | 1.08 (0.92, 1.26) | Reference | 1.21 (0.81, 1.81) | 1.20 (0.78, 1.84) | 1.04 (0.35, 3.07) | 3.02 (0.33, 27.72) | 1.79 (0.25, 12.98) |
| Males |  |  |  |  |  |  |  |
| Cases/total | 116 / 16660 | 74 / 10867 | 42 / 5793 | 31 / 4163 | 7 / 1153 | 2 / 381 | 2 / 96 |
| Model 1: basic | 1.02 (0.86, 1.21) | Reference | 0.98 (0.67, 1.43) | 1.01 (0.66, 1.54) | 0.80 (0.37, 1.74) | 0.67 (0.16, 2.75) | 3.25 (0.80, 13.24) |
| Model 2: model 1 + smoking + PA | 1.01 (0.85, 1.20) | Reference | 0.96 (0.65, 1.41) | 1.03 (0.67, 1.57) | 0.73 (0.33, 1.58) | 0.58 (0.14, 2.38) | 3.25 (0.80, 13.29) |
| Model 3: model 2 + alcohol intake | 1.01 (0.85, 1.20) | Reference | 0.95 (0.65, 1.40) | 1.09 (0.70, 1.68) | 0.62 (0.28, 1.39) | 0.37 (0.08, 1.77) | 3.25 (0.79, 13.26) |
| Model 4: model 3 + FIB4 score | 1.00 (0.83, 1.19) | Reference | 0.93 (0.62, 1.38) | 1.04 (0.66, 1.62) | 0.64 (0.28, 1.44) | 0.38 (0.08, 1.86) | 3.37 (0.82, 13.79) |
| Model 5: model 4 + BMI | 0.88 (0.71, 1.08) | Reference | 0.70 (0.45, 1.09) | 0.78 (0.48, 1.27) | 0.49 (0.21, 1.13) | 0.29 (0.06, 1.43) | 2.89 (0.70, 11.89) |
| Model 6: model 4 + WC | 0.88 (0.71, 1.08) | Reference | 0.70 (0.46, 1.09) | 0.78 (0.48, 1.27) | 0.50 (0.22, 1.14) | 0.29 (0.06, 1.42) | 2.69 (0.65, 11.12) |
| Model 7: model 4 + WHR | 0.96 (0.79, 1.17) | Reference | 0.86 (0.56, 1.30) | 0.96 (0.60, 1.53) | 0.60 (0.26, 1.36) | 0.35 (0.07, 1.72) | 3.17 (0.77, 13.04) |
| Model 8: model 4 + WHtR | 0.90 (0.73, 1.11) | Reference | 0.75 (0.48, 1.15) | 0.83 (0.51, 1.35) | 0.52 (0.23, 1.19) | 0.31 (0.06, 1.49) | 2.90 (0.70, 11.95) |
| Model 9: model 4 + BF% | 0.89 (0.72, 1.09) | Reference | 0.74 (0.48, 1.14) | 0.81 (0.50, 1.32) | 0.54 (0.24, 1.24) | 0.32 (0.07, 1.56) | 2.83 (0.69, 11.70) |

Basic Model: adjusted for sex, age, ethnicity, education and Townsend Deprivation Index. SLD: steatotic liver disease. MASLD: metabolic dysfunction associated steatotic liver disease. MetALD: metabolic dysfunction and alcohol related liver disease. ALD: alcohol related liver disease. PA: physical activity. BMI: body mass index. WC: waist circumference. WHR: waist-to-hip ratio. WHtR: waist-to-height ratio. BF%: body fat percentage.

### Supplementary table 5: Cross-sectional and prospective associations between liver fat severity and depression.

|  | Cross-sectional | | |  | Prospective | | |
| --- | --- | --- | --- | --- | --- | --- | --- |
|  | non-SLD | 5 < Liver fat < 10 | Liver fat ≥ 10% |  | non-SLD | 5 < Liver fat < 10 | Liver fat ≥ 10% |
| **Females + males** |  |  |  |  |  |  |  |
| Cases/total | 1923 / 26283 | 552 / 6162 | 386 / 4142 |  | 297 / 24360 | 73 / 5610 | 62 / 3756 |
| Model 1: basic | Reference | 1.39 (1.26, 1.54) | 1.40 (1.24, 1.57) |  | Reference | 1.43 (1.08, 1.89) | 0.76 (0.62, 0.92) |
| Model 2: model 1 + smoking + PA | Reference | 1.33 (1.20, 1.47) | 1.30 (1.15, 1.46) |  | Reference | 1.34 (1.01, 1.78) | 0.73 (0.60, 0.90) |
| Model 3: model 2 + alcohol intake | Reference | 1.34 (1.21, 1.48) | 1.31 (1.16, 1.47) |  | Reference | 1.35 (1.02, 1.78) | 0.74 (0.60, 0.91) |
| Model 4: model 3 + FIB4 score | Reference | 1.35 (1.22, 1.49) | 1.31 (1.16, 1.47) |  | Reference | 1.33 (1.00, 1.77) | 0.74 (0.60, 0.91) |
| Model 5: model 4 + BMI | Reference | 1.15 (1.03, 1.29) | 1.04 (0.91, 1.18) |  | Reference | 0.95 (0.70, 1.31) | 0.73 (0.59, 0.90) |
| Model 6: model 4 + WC | Reference | 1.15 (1.03, 1.29) | 1.03 (0.90, 1.18) |  | Reference | 0.98 (0.71, 1.34) | 0.59 (0.47, 0.75) |
| Model 7: model 4 + WHR | Reference | 1.26 (1.13, 1.40) | 1.18 (1.04, 1.34) |  | Reference | 1.21 (0.89, 1.64) | 0.63 (0.48, 0.83) |
| Model 8: model 4 + WHtR | Reference | 1.15 (1.03, 1.29) | 1.04 (0.91, 1.18) |  | Reference | 1.00 (0.73, 1.38) | 0.70 (0.57, 0.87) |
| Model 9: model 4 + BF% | Reference | 1.19 (1.07, 1.33) | 1.11 (0.97, 1.26) |  | Reference | 1.04 (0.76, 1.42) | 1.19 (0.88, 1.62) |
| **Females** |  |  |  |  |  |  |  |
| Cases/total | 1308 / 14766 | 283 / 2401 | 198 / 1627 |  | 170 / 13458 | 1.71 (1.17, 2.50) | 0.96 (0.92, 1.01) |
| Model 1: basic | Reference | 1.43 (1.24, 1.64) | 1.43 (1.22, 1.68) |  | Reference | 1.57 (1.07, 2.31) | 0.96 (0.92, 1.01) |
| Model 2: model 1 + smoking + PA | Reference | 1.37 (1.19, 1.57) | 1.34 (1.14, 1.58) |  | Reference | 1.59 (1.08, 2.33) | 0.96 (0.92, 1.01) |
| Model 3: model 2 + alcohol intake | Reference | 1.36 (1.19, 1.57) | 1.34 (1.14, 1.57) |  | Reference | 1.57 (1.06, 2.32) | 0.96 (0.92, 1.01) |
| Model 4: model 3 + FIB4 score | Reference | 1.38 (1.20, 1.59) | 1.37 (1.16, 1.61) |  | Reference | 1.11 (0.72, 1.71) | 0.96 (0.92, 1.01) |
| Model 5: model 4 + BMI | Reference | 1.15 (0.99, 1.34) | 1.05 (0.88, 1.26) |  | Reference | 1.14 (0.74, 1.76) | 0.96 (0.92, 1.01) |
| Model 6: model 4 + WC | Reference | 1.15 (0.99, 1.34) | 1.05 (0.88, 1.26) |  | Reference | 1.48 (0.97, 2.23) | 0.96 (0.92, 1.01) |
| Model 7: model 4 + WHR | Reference | 1.30 (1.12, 1.50) | 1.25 (1.05, 1.49) |  | Reference | 1.15 (0.75, 1.78) | 0.96 (0.91, 1.01) |
| Model 8: model 4 + WHtR | Reference | 1.16 (1.00, 1.35) | 1.06 (0.89, 1.28) |  | Reference | 1.23 (0.81, 1.87) | 0.95 (0.91, 1.01) |
| Model 9: model 4 + BF% | Reference | 1.22 (1.05, 1.42) | 1.15 (0.96, 1.37) |  | Reference | 1.71 (1.17, 2.50) | 0.96 (0.92, 1.01) |
| **Males** |  |  |  |  |  |  |  |
| Cases/total | 615 / 11517 | 269 / 3761 | 188 / 2515 |  | 109 / 10902 | 38 / 3492 | 30 / 2327 |
| Model 1: basic | Reference | 1.35 (1.16, 1.57) | 1.37 (1.15, 1.62) |  | Reference | 1.21 (0.81, 1.82) | 1.01 (0.96, 1.06) |
| Model 2: model 1 + smoking + PA | Reference | 1.28 (1.11, 1.49) | 1.25 (1.05, 1.49) |  | Reference | 1.17 (0.78, 1.77) | 1.01 (0.96, 1.06) |
| Model 3: model 2 + alcohol intake | Reference | 1.31 (1.13, 1.53) | 1.28 (1.08, 1.52) |  | Reference | 1.16 (0.77, 1.75) | 1.01 (0.96, 1.06) |
| Model 4: model 3 + FIB4 score | Reference | 1.31 (1.13, 1.53) | 1.24 (1.04, 1.48) |  | Reference | 1.13 (0.74, 1.73) | 1.01 (0.96, 1.07) |
| Model 5: model 4 + BMI | Reference | 1.16 (0.99, 1.36) | 1.01 (0.83, 1.24) |  | Reference | 0.89 (0.56, 1.41) | 1.01 (0.96, 1.07) |
| Model 6: model 4 + WC | Reference | 1.16 (0.99, 1.36) | 1.01 (0.83, 1.23) |  | Reference | 0.88 (0.56, 1.39) | 1.01 (0.96, 1.06) |
| Model 7: model 4 + WHR | Reference | 1.21 (1.04, 1.42) | 1.10 (0.91, 1.33) |  | Reference | 1.01 (0.65, 1.57) | 1.01 (0.96, 1.06) |
| Model 8: model 4 + WHtR | Reference | 1.15 (0.98, 1.35) | 1.00 (0.83, 1.22) |  | Reference | 0.92 (0.58, 1.46) | 1.01 (0.96, 1.06) |
| Model 9: model 4 + BF% | Reference | 1.16 (0.99, 1.36) | 1.05 (0.87, 1.28) |  | Reference | 0.94 (0.59, 1.49) | 1.01 (0.96, 1.07) |

Basic Model: adjusted for sex, age, ethnicity, education and Townsend Deprivation Index. SLD: steatotic liver disease. MASLD: metabolic dysfunction associated steatotic liver disease. MetALD: metabolic dysfunction and alcohol related liver disease. ALD: alcohol related liver disease. PA: physical activity. BMI: body mass index. WC: waist circumference. WHR: waist-to-hip ratio. WHtR: waist-to-height ratio. BF%: body fat percentage.

### Supplementary table 6: Subgroup analysis for the associations between liver fat (per 5%) and incidence of depression.

| Subgroups | Events/ total | HR (95%CI) | P for subgroup difference* |
| --- | --- | --- | --- |
| **Age** |  |  |  |
| 45-59 | 127 / 9229 | 1.04 (0.87, 1.24) |  |
| 60-69 | 169 / 14227 | 0.99 (0.85, 1.14) | 0.54 |
| 70+ | 118 / 10270 | 1.06 (0.86, 1.31) | 0.94 |
| **Townsend Deprivation Index** |  |  |  |
| 1^st^ fifth (least deprived) | 74 / 6822 | 1.11 (0.89, 1.40) |  |
| 2^nd^ fifth | 60 / 6770 | 1.10 (0.85, 1.43) | 0.42 |
| 3^rd^ fifth | 87 / 6777 | 1.08 (0.87, 1.34) | 0.45 |
| 4^th^ fifth | 83 / 6730 | 0.93 (0.73, 1.18) | 0.18 |
| 5^th^ fifth (most deprived) | 110 / 6600 | 0.95 (0.79, 1.15) | 0.35 |
| **Education** |  |  |  |
| Below secondary | 43 / 2206 | 1.19 (0.92, 1.54) |  |
| Lower secondary | 68 / 4367 | 1.05 (0.85, 1.30) | 0.74 |
| Higher secondary | 27 / 2023 | 1.09 (0.73, 1.64) | 0.36 |
| Vocational | 112 / 8696 | 1.00 (0.83, 1.21) | 0.30 |
| Higher education | 164 / 16434 | 0.95 (0.78, 1.15) | 0.18 |
| **Smoking** |  |  |  |
| Never | 219 / 21235 | 1.01 (0.88, 1.17) |  |
| Previous | 165 / 11312 | 1.05 (0.91, 1.22) | 0.93 |
| Current | 30 / 1072 | 0.84 (0.51, 1.39) | 0.34 |
| **Physical activity** |  |  |  |
| Low | 63 / 3155 | 1.29 (1.07, 1.54) |  |
| Moderate | 140 / 12044 | 0.99 (0.84, 1.16) | 0.47 |
| High | 144 / 13656 | 0.92 (0.73, 1.15) | 0.10 |
| **Body mass index** |  |  |  |
| ≤ 24.9 | 132 / 13838 | 0.79 (0.48, 1.31) |  |
| 25.0-29.9 | 175 / 14013 | 1.06 (0.91, 1.23) | 0.31 |
| ≥ 30.0 | 107 / 5817 | 1.00 (0.87, 1.15) | 0.44 |
| **SLD subtypes** |  |  |  |
| non-SLD | 279 / 24360 | 0.82 (0.40, 1.71) |  |
| MASLD | 100 / 7066 | 1.03 (0.88, 1.21) | 0.57 |
| MetALD | 25 / 1687 | 1.18 (0.88, 1.57) | 0.80 |
| ALD | 5 / 459 | 0.97 (0.37, 2.54) | 0.57 |

Results from a Cox model adjusted for age, sex, ethnicity, education, Townsend Deprivation Index, smoking, physical activity, alcohol intake, FIB4 score and body mass index. *: subgroup difference was tested by fitting interaction terms in the model. P values are relative to the first subgroup under each stratifying variable. SLD: steatotic liver disease. MASLD: metabolic dysfunction associated steatotic liver disease. MetALD: metabolic dysfunction and alcohol related liver disease. ALD: alcohol related liver disease. HR: hazard ratio. CI: confidence interval.
